# The importance of estradiol for body weight regulation in women

**DOI:** 10.3389/fendo.2022.951186

**Published:** 2022-11-07

**Authors:** Pilar Vigil, Jaime Meléndez, Grace Petkovic, Juan Pablo Del Río

**Affiliations:** ^1^ Reproductive Health Research Institute (RHRI), Santiago, Chile; ^2^ Arrowe Park Hospital, Department of Paediatrics, Wirral CH49 5PE, Merseyside, United Kingdom; ^3^ Unidad de Psiquiatría Infantil y del Adolescente, Clínica Psiquiátrica Universitaria, Universidad de Chile, Santiago, Chile; ^4^ Millennium Nucleus to Improve the Mental Health of Adolescents and Youths, Millennium Science Initiative, Santiago, Chile

**Keywords:** GLP-1, estrogens, body weight, menstrual cycle, mental health

## Abstract

Obesity in women of reproductive age has a number of adverse metabolic effects, including Type II Diabetes (T2D), dyslipidemia, and cardiovascular disease. It is associated with increased menstrual irregularity, ovulatory dysfunction, development of insulin resistance and infertility. In women, estradiol is not only critical for reproductive function, but they also control food intake and energy expenditure. Food intake is known to change during the menstrual cycle in humans. This change in food intake is largely mediated by estradiol, which acts directly upon anorexigenic and orexigenic neurons, largely in the hypothalamus. Estradiol also acts indirectly with peripheral mediators such as glucagon like peptide-1 (GLP-1). Like estradiol, GLP-1 acts on receptors at the hypothalamus. This review describes the physiological and pathophysiological mechanisms governing the actions of estradiol during the menstrual cycle on food intake and energy expenditure and how estradiol acts with other weight-controlling molecules such as GLP-1. GLP-1 analogs have proven to be effective both to manage obesity and T2D in women. This review also highlights the relationship between steroid hormones and women's mental health. It explains how a decline or imbalance in estradiol levels affects insulin sensitivity in the brain. This can cause cerebral insulin resistance, which contributes to the development of conditions such as Parkinson’s or Alzheimer’s disease. The proper use of both estradiol and GLP-1 analogs can help to manage obesity and preserve an optimal mental health in women by reducing the mechanisms that trigger neurodegenerative disorders.

## Introduction

Obesity, defined as a body mass index (BMI) ≥ 30, affects around 42% of adults in the United States (1). During the period 2017-2018, women had a higher prevalence of severe obesity (BMI ≥ 40 kg/m^2^) than men (11.5% vs. 6.9%, respectively) (1), though overall obesity prevalence rates were similar (42.1% and 43.0%, respectively).

Obesity is associated with a number of disorders that affect the reproductive system. Such disorders include: ovulatory dysfunction, such as found in polycystic ovary syndrome (PCOS); disorders of pregnancy, (e.g., preeclampsia, gestational diabetes, and recurrent pregnancy loss), endometriosis; and cancers (2, 3). There is growing concern about how the increasing obesity rate in adolescent women will impact their long-term health. The prevalence of obesity among adolescent girls (12–19 years) in 2015/2016 was 20.9% (4) and 3–11% of these obese adolescent girls had PCOS (5, 6). This may be explained by the fact that obesity and related comorbidities, such as insulin resistance, alter the functioning of the hypothalamic–pituitary–ovarian axis, decreasing ovarian responsiveness to gonadotropin stimulation (3, 7). Insulin also stimulates follicular growth through its action at the theca cells (8). This causes disorganized follicular growth and increases ovarian production and secretion of testosterone (9). Ultimately, this can, in turn, affect ovulation. Amongst PCOS patients, those who are obese are most at risk of insulin resistance (10). By contrast, only half (50%) of normal weight PCOS patients are insulin resistant (9).

**Figure 1:**
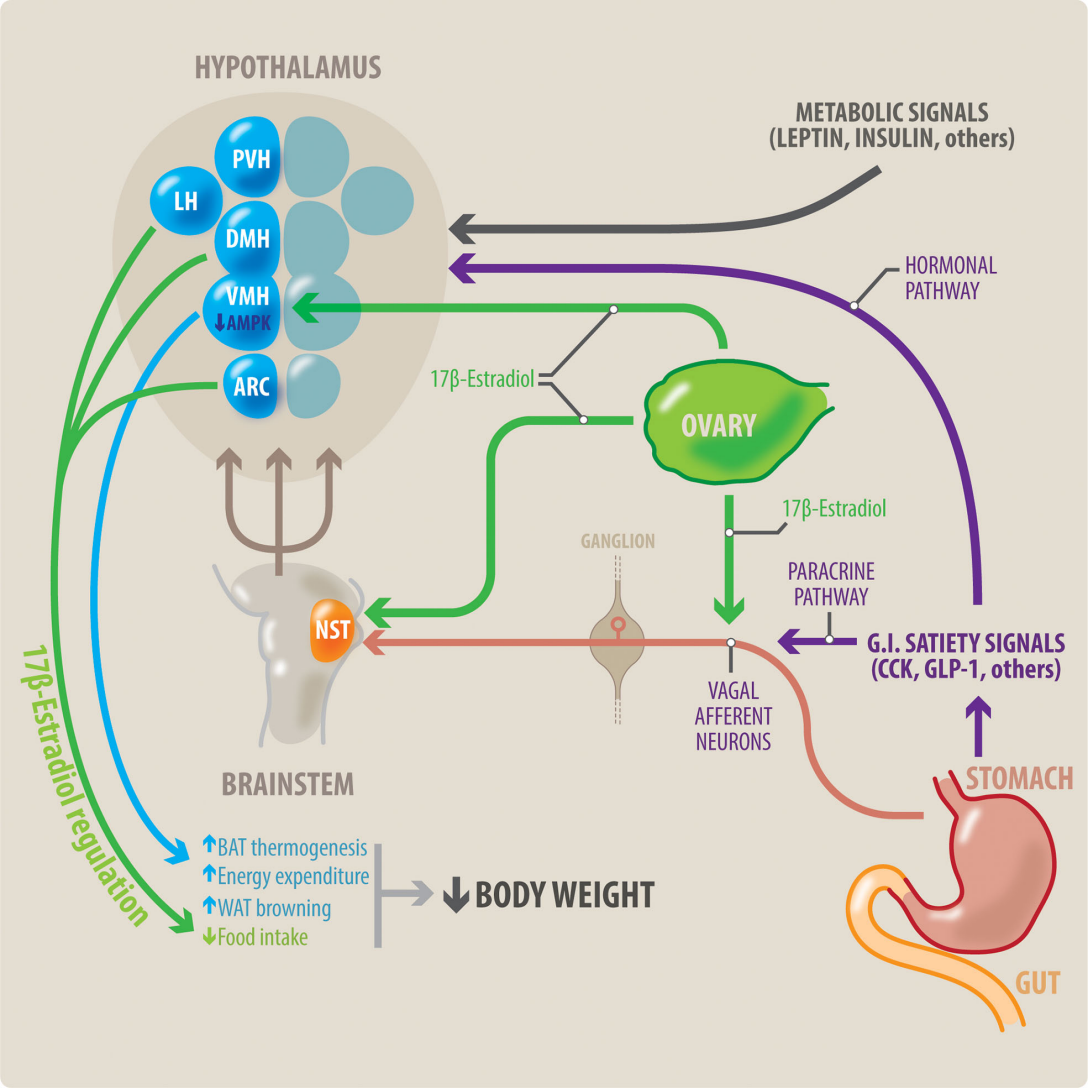
Potential interaction between meal-related gastrointestinal signals and estradiol on control of the body weight in women. Meal-related gastrointestinal signals (CCK, GLP-1, others) act through a paracrine-neuronal pathway (shown in purple and red). These meal-related gastrointestinal signals act paracrinally upon vagal afferent neurons (VAN). The VANs activate secondary neurons located in the NTS, in the brainstem. The NTS integrates a variety of peripheral signals, and in turn activates tertiary neurons located in different nuclei in the hypothalamus. These hypothalamic nuclei control feeding behavior. Circulating estradiol (shown in green) modulates the responsiveness to these gastrointestinal satiety signals by acting on all levels of this paracrine-neuronal pathway: The VAN, NTS and the hypothalamic nuclei. Gastrointestinal satiety signals also act directly upon the hypothalamic nuclei through a hormonal pathway (shown in purple). Additionally, estradiol (green) has a direct anorexigenic effect at the level of the hypothalamic nuclei (PVH, LH and ARC), thereby reducing food intake. Metabolic signals such as insulin and leptin also influence centers in the hypothalamus to regulate body weight. Brown adipose tissue (BAT) thermogenesis contributes to regulation of body weight by increasing energy expenditure. Estradiol acts all three points of the VMH-SNS-BAT pathway to increase thermogenesis. Within the VMH hypothalamic nucleus, estradiol acts by inhibiting AMPK. Thus, estradiol increases energy expenditure by increasing BAT thermogenesis, and WAT browning. This, in combination with estradiol’s effects to decrease food intake, can result in weight loss. NST, nucleus of the solitary tract; DMH, dorsomedial hypothalamus; LH, lateral hypothalamus; PVH, paraventricular hypothalamus; ARC, arcuate nucleus; VMH, ventromedial hypothalamus; BAT, brown adipose tissue; WAT, white adipose tissue.

The reproductive system modulates body weight regulation. Food intake is known to change during the menstrual and/or estrous cycle, with women significantly reducing their food intake in the peri-ovulatory period (11–13). Therefore, in principle, ovulatory dysfunction may increase the risk of obesity, as women will lack this usual period of “reduced appetite”. This link between reproductive function and body weight control is largely mediated by the female sex steroid hormones, particularly estradiol and progesterone. In general, estradiol regulates homeostatic nutrition in women by decreasing food intake and increasing energy expenditure (13) (**Figure 1**). Female reductions in food intake during the peri-ovulatory period are a consequence of the anorectic action of estradiol. Estradiol acts at the level of the cortex, hypothalamus and brainstem (14). The anorectic and thermogenic effects of estradiol can be direct, through genomic and non-genomic mechanisms, or indirect, through activation of peripheral mediators such as cholecystokinin (CCK), insulin, leptin and GLP-1.

A reduction in estradiol levels, as found in the menopause, would therefore be expected to result in increased food intake (with the estradiol activity lost). Thus, a loss of estradiol post-menopause, may contribute to the development of obesity, and systemic and cerebral insulin resistance (15). Insulin resistance and T2D, both of which are associated with obesity and ovulatory dysfunction, cause abnormalities in the proper functioning of the central nervous system (CNS). Indeed, both are linked to neurodegenerative disorders (15, 16). Estradiol and GLP-1 (and its analogs) have been proposed as novel therapeutic approaches to restore not only body weight in women but also to prevent the development of neurodegenerative disorders (17, 18).

This review describes the physiological and pathophysiological mechanisms that govern the actions of estradiol on food intake and energy expenditure during the menstrual cycle (13). We highlight how GLP-1 and estrogen are thought to have synergistic effects and summarize recent work on the use of GLP-1 conjugates as agents to manage obesity, T2D and central insulin resistance. Finally, we consider potential future uses of estradiol and GLP-1 conjugates in protecting against cerebral insulin resistance, and resultant neurodegenerative disorders.

## Menstrual cycle and appetite control: Implications for weight regulation

The CNS, particularly the hypothalamus, plays a key role in homeostatic feeding. Brain nuclei such as the nucleus of the solitary tract (NST), the arcuate (ARC), the paraventricular region of the hypothalamus (PVH), control meal size of and modulate feelings of satiety. Additional brain regions are also involved in feeding. Such regions include: the primary and secondary taste regions (insula and orbitofrontal cortex); as well as the hippocampus; and cognitive control regions (dorsolateral prefrontal cortex, inferior frontal cortex and cingulate cortex) (19, 20). Eating behavior depends on the simultaneous operation of these homeostatic pathways together with a more flexible non-homeostatic pathway. The non-homeostatic pathway differs between individuals because of variations in hormonal status, epigenetic markers and personal experiences. Evidence from human and animal studies indicates that food intake fluctuates during the menstrual cycle, because gonadal steroid hormones (estradiol and progesterone) are key regulators of energy uptake.

There is strong evidence for a link between the menstrual and/or estrous cycle and appetite. For example, in laboratory studies, ovariectomized female rats increase their food intake. Their food intake can be normalized by the administration of physiological doses of -estradiol but not progesterone (11, 13, 21, 22). Other behavioral studies in rats have also demonstrated that estradiol controls meal size (23). In clinical studies, food intake is lower in the periovulatory phase and greater in the early follicular and luteal phases (11, 24, 25). The periovulatory decrease in food intake coincides with a surge in circulating estradiol levels and is the result of decreased meal size rather than decreased meal frequency (26, 27). The types of foods eaten also change with the menstrual cycle. Food cravings and binge eating of specific food items are reported more frequently by women in the luteal phase (28–30). Most (29–38) though not all (39–41) studies, have shown increased caloric intake in the luteal phase. The specific macronutrient composition of the increased calories consumed in the luteal phase varies, but most often results from either increased fat (32, 35, 38) or carbohydrate intake (35, 36, 38). Intake of sweet foods also decreases in the peri-ovulatory period (29, 42) and protein intake increases in the luteal phase (43). Orosensory stimuli affect organism’s selection and preference for particular foods. Estradiol levels affect how women psychologically perceive food (13, 44, 45). Across the menstrual cycle, neuronal responses to images of food change (46–49). When a subject is presented with high energy-food pictures in the periovulatory (as compared with the luteal phase), the brain areas linked to food intake show increased responsiveness (44). Dopaminergic reward activity to high energy foods is enhanced in the periovulatory phase (50, 51). Although the evidence is still controversial, the odor detection threshold may vary across the menstrual cycle. The threshold appears lower during the ovulatory and luteal phase (when estradiol levels are high) and higher during menstruation and early follicular phase (when estradiol levels are low) (44, 47, 52). Interestingly, it seems that the usual cyclical change in food intake is absent in anovulatory cycles (33, 43). This is explained by the absence of the estradiol´s rise and fall, impacting both appetite and ovulation. Anovulatory cycles can be associated with either low or, by contrast, constantly elevated estradiol levels (53). Both estradiol states could be linked to increased appetite. Evidently, a low estradiol level may be insufficient to trigger the usual anorectic effects. However, it may also be that at constant, high levels of estradiol (as in anovulatory cycles, or in hormonal preparations), its anorexic effects are blunted.

## Menstrual cycle and eating disorders

In addition to influencing food intake in healthy states, estradiol and progesterone have also been implicated in the etiology and expression of eating disorders (54–56). Eating disorders are one of the most sex differentiated forms of psychopathology, with the female-to-male ratio ranging from 4:1 to 10:1 (57). Binge eating and emotional eating are significantly higher during the mid-luteal and pre-menstrual phases of women’s menstrual cycle as compared to the follicular/ovulatory phases (54, 55, 58, 59). Progesterone levels are positively associated with increased binge eating across the menstrual cycle (54, 55). Whilst physiological levels of estradiol are inversely associated with binge eating, it appears that abnormally high levels of estradiol are actually positively associated with binge eating and emotional eating (13). Importantly, in all previous studies, hormonal effects on binge eating and emotional eating were independent of covariates that could also change across the menstrual cycle, such as negative affect and body mass index (BMI) (54, 55, 59, 60). Laboratory studies suggest estradiol may act on serotonergic neurons to inhibit binge eating (61) and this effect is partially mediated by insulin. Thus, increased insulin resistance may decrease the serotonergic neurons responsiveness to estradiol. This in turn, may increase the risk of binge eating. Even in healthy women, increased insulin resistance has been reported during the luteal phase of the menstrual cycle in healthy women (62). This could partially explain the differences in eating behavior observed across the menstrual cycle. Evidence suggests that women with an eating disorder may display differential insulin sensitivity to the changes in ovarian hormone levels (60, 62, 63).

It is interesting to speculate as to whether progesterone-only contraceptives could indirectly alter insulin sensitivity (64, 65). Many women receive progesterone only medications. Such medications disrupt the normal ovulatory process. Estradiol levels are therefore decreased (66). This reduction in estradiol and its insulin sensitizing effects could potentially decrease food intake and body weight in some women (67).

## Gonadotropins and adiposity

Although, gonadotropin hormone analogs have been used clinically for decades in assisted reproductive therapies and in the treatment of various infertility disorders (68), novel applications of gonadotropins targeting extra-gonadal tissues (69), especially adipose tissue and liver are emerging (70–73). Recent evidence suggests a possible role for FSH in regulating lipid metabolism and fat accumulation.

Postmenopausal women have low estradiol, elevated FSH, concomitant bone loss, and increased body fat). The rise of FSH at menopause in response to ovarian failure has been associated with menopausal adiposity (70) and hepatic steatosis (72) in women. Using mouse models, high circulating FSH has been confirmed as a major contributor to gonadectomy-induced obesity (70–72). hese findings suggested that FSH, as well as low estradiol, are potential targets for controlling fat accumulation and treating obesity.

In an ovariectomized mouse model, an antibody (for humans and mice) to FSHβ (was initially found to inhibit bone resorption and stimulate bone synthesis (61). Later, the same antibody was found to increase BAT thermogenesis and prevent (71) ovariectomy-induced weight gain and fat accumulation in mice (74). Mechanistically, FSH vaccination treatment inhibited lipid biosynthesis by inactivating PPARγ adipogenic signaling pathway and simultaneously enhancing adipocyte thermogenesis *via* upregulating UCP1 (uncoupling protein 1) expression in both visceral and subcutaneous adipose tissues (74).

Although evidence that FSH is a key factor in fat accumulation is robust, this is so far applicable only to some rodent models. Thus far, there are contradictory findings in both human and other rodent studies.

## Estradiol pathways in the regulation of body weight

### Estradiol mechanisms of action

As mentioned earlier, the hypothalamus integrates most of the neural and humoral afferent signals coordinating energy intake and expenditure (19). Among hypothalamic nuclei, the effects of the ARC on appetite are well-studied. The ARC contains two main types of neuronal systems: appetite-suppressing POMC neurons (75, 76); and, appetite-stimulating NPY/AgRP neurons (75, 77). Both these neuron systems express estrogen receptors: ERα is predominantly expressed in the POMC (proopiomelanocortin) neurons, while both ERα and ERβ are present in neuropeptide Y (NPY) and agouti-related protein peptide (AgRP) neurons (78, 79).

POMC is a precursor polypeptide, which after being released, is cleaved into smaller active peptides. One such peptide (α-MSH) is particularly important for appetite control. α-MSH is most known for its role in melanin production in skin through the activation of MC1R. However, it has an anorexigenic effect when it activates other receptors (MC3R and MC4R) located in the ARC and lateral hypothalamus (LH) (75). Indeed, mice that are deficient for MC4R or POMC are characteristically obese due to hyperphagia (80, 81). Estradiol has an anorexigenic effect by increasing POMC neuronal activity. Estradiol’s action on these neurons is both direct and indirect. Estradiol indirectly increases POMC activity through its effects on NPY/AgRP neurons (82, 83). Estradiol inhibits NPY/AgRP neurons primarily through glutamate and β-endorphin release (84). NPY and AgRP antagonize the action of α-MSH on MC3R and MC4R, thus having an orexigenic effect (85). Mice that overexpress AgRP are hyperphagic and obese. MC4R is also known to be important for appetite control in humans. MC4R mutations are the most frequent cause of monogenic obesity in humans (75).

Estradiol also decreases appetite directly by increasing anorexigenic gene expression in POMC neurons and decreasing the expression of orexigenic genes in NPY/AgRP neurons (84, 86). Interestingly, as female rats get older, these genes become less responsive to estradiol (87). Estradiol also has non-genomic effects. These effects are mediated by: Gq-mER (Gq-coupled membrane ER); GPER (G-protein-coupled estrogen receptor); and by ERα and ERβ present in the plasma membrane (88–91). Gq-mER is present in the hypothalamus and its expression is restricted to NPY/AgRP neurons where it decreases neuronal activity (82, 83, 92). GPER is expressed in a number of other hypothalamic nuclei, such as the PVH, the supraoptic nucleus and the medial preoptic area (mPOA) (93). GPER deficiency causes increased adiposity, insulin resistance, and metabolic dysfunction in mice (90).

## Estradiol, AMPK and thermogenesis

Estradiol also affects weight regulation by impacting thermogenesis. Thermogenesis is the dissipation of energy through heat production. This increased energy expenditure contributes to weight loss. Thermogenesis may occur through both shivering and non-shivering mechanisms (94). Brown adipose tissue (BAT) is a specialized fat depot characterized by increased energy expenditure and heat production (95). Its expansion and/or activation can protect against diet-induced obesity. The classical thermogenesis pathway revolves around the sympathetic nervous system-catecholamine-uncoupling protein 1 axis. UCP1 is a proton channel which allows dissipation of the proton gradient across the mitochondrial matrix, without adenosine triphosphate (ATP) production. This dissipation generates energy in the form of heat. Activation of the sympathetic nervous system (SNS) releases catecholamines (e.g. norepinephrine) which increase UCP1 activity. Centrally, several hypothalamic regions, most especially the ventromedial hypothalamus (VMH), are known to regulate this pathway. Electrical or pharmacological stimulation of this nucleus increases BAT thermogenesis (96–101). Estradiol modulates thermogenesis at three points on this VMH-SNS-BAT pathway: 1) through its effects on the VMH nucleus 2) through its effects on SNS signaling 3) directly through its effects on BAT.

Within the VMH nucleus, AMP-activated protein kinase (AMPK) appears to be a key mediator of estradiol’s effects. AMPK is a so-called ‘cellular energy sensor’ (102, 103). It senses the ADP:ATP and AMP:ATP ratios and alters ATP production as a cell requires (104). AMPK activation in the VMH decreases energy expenditure (105–107) and AMPK inhibition increases energy expenditure (**Figure 1**). Animal models have shown that estradiol increases energy expenditure through increased thermogenesis and lipolysis of BAT. Estradiol-induced BAT thermogenesis and its consequent body weight loss can be prevented by activation of AMPK in the VMH (108). This suggests that estradiol may increase thermogenesis by the inhibiting AMPK at the VMH nucleus of the hypothalamus. AMPK also acts as an important mediator for other peripheral modulators of thermogenesis (109). Such modulators include thyroid hormone, GLP-1, and leptin (109, 110). AMPK may also mediate other effects of estrogens, e.g. on glucose homeostasis (111).

Estradiol acts at the second point of the VMH-SNS-BAT pathway by increasing norepinephrine turnover, thus increasing non-shivering thermogenesis (112, 113). It also acts on BAT tissue directly, though interestingly not on UCP1 (114–116).

It is important to mention that BAT is a specialized fat depot characterized by increased energy expenditure and heat production (95). Its expansion and/or activation can protect against diet-induced obesity. Beige adipocytes that share some common characteristics with brown adipocytes such as high mitochondria content and uncoupling protein 1 (UCP1) expression can be induced in white adipose tissue (WAT). This process is called WAT browning (117).

## Interactions between estrogens and peripheral feedback signals controlling appetite

Thus far we have considered estradiol’s direct effects on the central control of appetite. Estradiol’s central effects on appetite are also modulated by a number of other peripheral signals (**Figure 1**). These signals include peptides secreted by the gastrointestinal tract (CCK, GLP-1), the pancreas (glucagon and insulin) and adipose tissue (leptin).

## CCK interaction with estradiol in satiety control

Cholecystokinin (CCK) is a key controller of meal-ending satiation in animals and humans (118–121). CCK is particularly known for its local effects in the gastrointestinal system. It is produced by cells lining the duodenum and its name derives from its effect on the gallbladder, causing it to contract and release bile into the intestine. However, further work has since revealed that CCK is also important for satiety. Indeed, CCK has moved from being considered a local gut hormone, to being recognized as an almost ubiquitous chemical messenger (122). We will consider its effect on peripheral and central satiety control mechanisms.

Peripherally administered CCK reduces food intake (123). This effect is mediated by the vagus nerve. CCK increases vagal excitability (124–127). The vagus in turn stimulates second order neurons in the NST (128, 129), located in the medulla of the brainstem (**Figure 1**). NST plays a key role in appetite. This is perhaps unsurprising given that it integrates gustatory and visceral input from cranial nerves VII, IX, and X (130). The NST, in turn, signals to a number of other brain centres that impact satiety. In summary, CCK activates vagal fibres, which in turn activate NST neurons, inducing a feeling of satiety.

Evidence for estradiol’s interaction with CCK comes from studies of ovariectomized rats. In ovariectomized rats, subcutaneous replacement of estradiol potentiates suppression of total food intake induced by CCK (22, 131, 132). Furthermore, estradiol potentiates endogenous CCK-induced suppression of food intake in both hormone-replaced ovariectomized and estrous control females (133–136). Estradiol modulates vagal nerve reactivity and NST activity. In ovariectomized rats, replacement of estradiol in this nucleus reduces food intake and this effect is blunted by co-administration of an ERα antagonist (137, 138). Estradiol augments the density of axonal projections and the excitability of vagal afferent neurons (139). The sensitivity of the vagal nerve to estradiol fluctuates through the estrous cycle, as ERα expression changes in response to circulating estradiol levels (140).

Thus far, we have focused on peripherally produced CCK. However, as previously indicated, CCK is now known to be produced almost ubiquitously, including in the brain. CCK producing neurons are known to be important for appetite control. Estradiol has been shown to impact this central CCK expression. For example, administering physiological doses of estradiol dramatically increases CCK mRNA levels in the posterodorsal medial amygdaloid nucleus (MeApd) and in the central part of the mPOA. These regions are part of the limbic-hypothalamic circuit (141). There is evidence to suggest that, as in the NST, CCK and estradiol also act synergistically in the limbic-hypothalamic circuit. This is indicated by CCK expression in pertinent brain regions (e.g. hypothalamus) changing with estrous cycle phase in rats. Specifically, CCK expression is highest during the pro-estrous phase when plasma estradiol levels are at their highest (132, 133, 142). Taken together, it is likely that estradiol’s anorexigenic effects are due not only to estradiol’s direct effects on appetite, but also due to its interaction with other molecules, such as CCK.

## Leptin

Leptin is an adipocyte-derived hormone that reflects energy storage (143). In normal conditions, leptin prevents body weight gain by suppressing feeding (144, 145) and increasing energy expenditure (146–148). In general, leptin down-regulates orexigenic peptides, and up-regulates anorexigenic peptides, leading to a reduction in food intake. In particular, leptin modulates the signals for satiety found in the ARC. For example, when leptin levels are reduced, POMC expression is also reduced and NPY expression is increased (149). Furthermore, as mentioned, POMC is a precursor for α-MSH, which helps control appetite. α -MSH antagonists antagonize leptin’s anorexigenic effect (150). It must be remembered that estradiol exerts some of its direct effects on satiety through these same neuronal populations. Interestingly, both estradiol and leptin receptors colocalize in kisspeptinergic neurons, which are considered to be the link between nutrition (metabolism) and reproduction (ovulatory function) (151).

These neurons are present in the ARC, VMH, and POA (152). Such co-localization raises the question: might leptin and estradiol interact centrally? The answer appears to be yes. In the pro-estrous (high estradiol) phase of the estrous cycle, estradiol increases leptin mRNA expression and serum leptin levels (153). Furthermore, it has been shown that physiological high estradiol levels correlate with increased leptin sensitivity and that reduced leptin sensitivity after oophorectomy can be restored with estrogen treatment (154). Deletion of leptin receptors in vagal afferent neurons disrupts estrogen signaling, body weight, food intake and hormonal controls of feeding in female mice (140). Thus, estradiol and leptin interact to inhibit nutrient uptake. Clinically, it is interesting to consider how this may alter the usual pattern of cyclical feeding changes in obese women. Leptin resistance, commonly found in obese women, is likely to blunt the peri-ovulatory anorexigenic effect of estradiol. It is already known that leptin resistance also disrupts ovulatory function by inhibiting the kisspeptinergic system (151). This ovulatory dysfunction leads to abnormal estradiol values, thus likely further affecting physiological body weight regulation.

## Insulin

Insulin affects energy balance regulation (155). Basal plasma insulin concentrations are proportional to body adiposity (156). Insulin’s secretion and synthesis are affected by a number of genetic, environmental and epigenetic factors. For example, dietary choices impact insulin secretion and synthesis (157). Insulin, like leptin, stimulates anorexigenic pathways, thereby causing reduced food intake. Insulin receptors are expressed on hypothalamic neurons, predominantly in ARC (158). Insulin affects appetite by reducing the expression of NPY neurons in the ARC (159). Again, this is where estradiol also exerts a number of direct effects on appetite, thus raising the possibility of estradiol-insulin interactions to alter food intake.

Furthermore, estradiol and insulin are thought to interact at the peripheral level. For example, estradiol may protect against the development of metabolic syndrome by impacting insulin sensitivity. The post-menopausal drop in estradiol levels is thought to explain, at least partially, the increase in metabolic disorders in post-menopausal women (160–163). In support of this hypothesis, hormone replacement therapies (HRT) that include estradiol have been shown to improve insulin sensitivity and lower blood glucose levels (164–166). This improvement in insulin sensitivity reduces the incidence of diabetes in postmenopausal women (167–169). Support for estradiol altering insulin sensitivity has also been reported in rodents, since estradiol deficient animals are more likely to develop insulin resistance (170, 171).

The clinical implications of this interaction extend beyond hypo-estrogenic states (e.g. post-menopause). A number of women have a hyper-estrogenic state. This can be as a consequence of endocrinopathies (such as PCOS) or simply of their life stage (e.g. perimenopause). Supra-physiological concentrations of estradiol induce a decrease in the expression of insulin receptors, thereby contributing to the development of insulin resistance (172, 173). High doses of estradiol also significantly decrease the amount of insulin receptors and the insulin receptor substrate 1 (IRS-1) levels in muscle and adipose tissue *in vitro* (174). These changes induce a greater release of intracellular calcium given the high concentrations of estradiol, inducing a greater release of insulin into the bloodstream, contributing to sustained hyperinsulinemia. Over time, insulin resistance contributes to the development of obesity, diabetes and cardiovascular diseases, and sustained hyperinsulinemia contributes to the generation and/or maintenance of ovulatory dysfunction (175, 176).

Overall, this evidence suggests that abnormally high or low levels of estradiol can both lead increased insulin resistance in the brain and peripheral tissues. This means that insulin resistance may be more likely to develop during hyper-estrogenic periods of a woman’s life. Such periods include adolescence, pregnancy and the perimenopause. Women could be more at risk of weight gain during these stages.

## GLP-1

GLP-1 is secreted by the pancreas and by intestinal L-cells in response to glucose-induced insulin release (177, 178). It also reduces glucagon secretion in response to a nutrient load (179). Whilst it has a paracrine or endocrine role in the periphery, centrally GLP-1 is an important neuroendocrine agent. GLP-1 is produced in various brain regions including the hypothalamus, the hippocampus, the hindbrain, and the mesolimbic system (180). Both human and animal studies have demonstrated that GLP-1 contributes to the physiological control of appetite and meal size (108, 181–186). Suppression of GLP-1R expression in NST neurons in animal models causes an increase in food intake due to an increase in meal size (187). By contrast, central injections GLP-1R agonists cause a reduction in food intake (188). This anorexic response is largely mediated by neuronal areas within the hypothalamus and brainstem (189–193). Many of these areas are also the ones where estradiol acts to control appetite (194–197). As mentioned earlier, estradiol impacts vagal nerve fiber excitability and density. In the same way, GLP-1R expression in vagal afferent neurons has also been found to be important for affecting food intake and meal size (198). Such co-localization in the sites of GLP and estradiol activity raises the possibility of estradiol and GLP-1 interacting to alter food intake. Support for this hypothesis comes from the finding that, in ovariectomized rats, estradiol replacement enhances peripheral GLP-1 induced suppression on food intake (199, 200).

## Estradiol, brain insulin sensitivity and resistance

The activation of estrogenic pathways exerts a neuroprotective effect in the CNS through four different mechanisms [reviewed in (53)]. Low estradiol values may be found in obese women with ovulatory dysfunction and in women during the peri-menopausal and post-menopausal periods. This means that such women lose the neuroprotective effect of estradiol. Briefly, estradiol improves neuronal survival through:

1. *Activation of anti-apoptotic and cell survival pathways* (90, 201). Estradiol promotes anti-apoptotic pathways by enhancing the transcription of anti-apoptotic genes such as B-cell lymphoma 2 (BCL2) (202) and inactivating pro-apoptotic proteins such as BAD (BCL2 associated agonist of cell death) (203, 204).
2. *Regulation of bioenergetics systems.* Estradiol increases glucose availability and ATP production in neuronal mitochondria (205). It does this by increasing the number of glucose transporters, glucose uptake and the activity of glycolytic enzymes in aerobic glycolysis (201). It also helps ensure neurons meet their high energy demands appropriately.
3. *Regulation of neurogenesis*. Estradiol stimulates proliferation of neural progenitor cells in a time- and dose-dependent manner (201, 206).
4. *Increased cell survival through protection against free-radical damage*. Estradiol reduces oxidative damage and its consequent apoptotic process (205).

Mitochondria are commonly considered the cellular powerhouse sustaining life. Mitochondria produce ATP, enabling stress adaptation for survival. During the production of ATP, the transport of electrons generates reactive oxygen species (ROS) that damage macromolecules, such as mitochondrial DNA, proteins and lipids. This macromolecular damage can contribute to mitochondrial stress. Estradiol regulates mitochondrial morphology and function (207). Estrogens and androgens protect mitochondria against the degenerative effects that occur with aging (208). Estradiol inhibits the activation of cell death caused by ROS (209). When estradiol levels start to decline as women age or they transit to menopause, the protective effect of estradiol is lost (210). As a consequence, when estradiol levels are reduced, ROS levels increase and cause mitochondrial dysfunction. Mitochondrial dysfunction is associated with an imbalance between pro- and anti-oxidants (210). As this dysfunction worsens, significant mitochondrial damage can occur. This damage triggers tissue events associated with cellular senescence as loss of replicative capacity. In the brain, neuronal damage and alteration of cognitive processes occur. Mitochondrial dysfunction occurs in all individuals with age (211). However, the post-menopausal drop in estradiol levels exacerbates this mitochondrial dysfunction. Sex hormone treatment during menopause transition helps reverse the deleterious effects of the drop in estradiol (212).

Insulin also has neuroprotective effects. Insulin action has been found to improve visual and spatial episodic memory, working memory, declarative memory, and learning processes (reviewed in (16)). The neuronal mitochondrial dysfunction that follows a drop in estradiol levels in women causes insulin insensitivity and eventually brain insulin resistance to develop (16, 213, 214).

A proper brain insulin action has been shown to improve mood and counteract cognitive dysfunction in dementia (215). Patients with chronic diabetes are more likely to suffer cognitive impairment, and a number of neurodegenerative disorders, including Alzheimer´s Disease (AD), Parkinson’s Disease and other forms of dementia. All these disorders share the following pathophysiological features: amyloid β accumulation, tau hyperphosphorylation, cerebral vasculopathy, inflammation, and oxidative stress in the CNS. These features are indicative of impaired insulin sensitivity in neurons and glial cells (15, 216–218). The term “Type III diabetes” has been proposed to describe AD that may develop from glucose and insulin dysregulation at the CNS (213, 219).

Finally, is important to highlight that physiological brain insulin sensitivity has also been identified as a predictor of successful weight loss (220). Evidence shows that a high cerebral sensitivity to insulin is related to weight loss (220). Conversely, cerebral insulin resistance leads to increased body weight and obesity. Reduced cerebral insulin sensitivity disrupts the neural controlling food intake. This result in overeating and weight gain (221).

## GLP-1 analogs and weight regulation

Weight control is key to combatting obesity and T2D. Recently, attention has turned to using GLP-1 analogs, or GLP-1/glucagon co-agonism to treat these disorders [for review see (222–228)]. For example, liraglutide is a once-daily, subcutaneously administered, GLP-1 receptor agonist (229–232). Results obtained from clinical trials show that it can aid weight loss (229, 230, 233). Indeed, a meta-analysis suggested that GLP-1 agonists may improve weight loss and insulin resistance in obese/overweight women than metformin does (234). Liraglutide has both peripheral and central effects. Peripherally, liraglutide delays gastric emptying, thus increasing the production of other peripheral satiety signals (235). Central administration of liraglutide in laboratory studies results in weight loss through decreased food intake (188, 236). This is mediated by the ARC, PVH, and LH hypothalamic nuclei (188, 236). Liraglutide action in the VMH also increases thermogenesis by increasing UCP1 expression in BAT and WAT (white adipose tissue) (188). Like estradiol, liraglutide inhibits AMPK activity in VMH neurons (188) and alters SNS activity as part of the VMH-SNS-BAT pathway. This is shown by the fact that catecholamine (specifically β3-AR) antagonists block the liraglutide-induced increase in UCP1 levels in BAT and WAT (188, 236).

## Estrogens for weight control

Post-menopause, women tend to gain weight. This weight gain is often attributed to aging in general as well as to hormonal changes. As discussed earlier, estradiol levels affect mitochondria. Mitochondria are considered to be the cellular ‘hub’ of aging (210). Estradiol receptors are located in both the inner and outer mitochondrial membranes, as well as in the cell plasma membrane, cell cytoplasm and nucleus (209). The menopausal drop in estradiol will thus affect the process of ATP synthesis and so will alter cellular metabolic pathways. Mitochondrial dysfunction will induce cellular senescence, most especially in brain, adipose and muscle tissues, thus affecting cellular metabolic control, fat distribution and weight gain.

In premenopausal women, there is significant interest in whether supra-physiological concentrations of estradiol, as found in combined hormonal contraceptives (CHC), impact women’s weight (237). This is complicated by the fact that combined contraceptives contain not only estrogens, but also progestins. Thus far, a 2014 Cochrane review found that there was insufficient evidence to determine the effect of CHCs on weight (237). The review found only four trials comparing CHCs with placebo, of which only one followed patients for over a year (238), whilst the remaining three followed weight changes over only 6-9 cycles. A review in which progestin-only contraceptives were studied highlighted the importance of the follow-up period, since longer follow-up periods (2-3 years) showed twice the degree of weight gain as compared to shorter studies (1 year) (239). Thus, the Cochrane review may have been somewhat limited by not just the number of women studied but the duration of clinical trials.

In the post-menopausal population, a 2005 Cochrane review found no effect of estradiol (opposed or unopposed) on women´s weight (240). However, since this review, a subsequent study (KEEPS study) found that BMI increased significantly less (by 1.09kg/m^2^) in women on HRT rather than placebo (241, 242). Such findings are in keeping with a 2009 HRT study (243) finding that women randomized to HRT gained less weight than those on placebo.

The evidence shown above reinforces the fact that estradiol as a main regulator of mitochondrial function would be an essential factor for healthy weight maintenance in women.

## GLP-1 and estradiol conjugates

Estradiol and GLP-1 conjugates share many effects and common pathways in weight control (244). Laboratory studies investigating a potential synergistic interaction between estradiol and GLP-1 have given inconsistent results. One study showed little synergy between exogenously administered labile estradiol-GLP-1 conjugates (245). However, another study noted sex- differences, which could be related to sex steroid levels, in the response to GLP-1 agonists (246). The main differences in GLP-1 activity that have been found between males and females are: i) increased weight loss caused by the GLP-1 agonist, liraglutide, in women as compared with men (247); ii) more immediate increases in GLP-1 levels immediately post-exercise in women during the follicular-phase than in men (248) and iii) increased reward circuits activation following GLP-1 administration in female rats as compared to male rats. Further work is needed to investigate whether it is estradiol that mediates these sex differences in GLP-1 activity. Endogenous GLP-1 levels have been shown to be lower in the follicular phase compared with the luteal phase (27). This is thought to be due to slower gastric emptying during the follicular phase (27). The luteal phase of the menstrual cycle has higher levels of estradiol and progesterone. It would be interesting to observe whether exogenous GLP-1 is more efficacious in women when administered during this hormonal phase as compared with the follicular phase. If so, then perhaps therapeutic GLP-1 could be given more infrequently, but synchronized to women’s hormonal cycles.

Concomitant use of GLP-1 and estradiol conjugates has been primarily limited by the oncogenic and gynecological side-effects that have been attributed to estrogens. Furthermore, weight loss associated with GLP-1 administration alone often fails to meet the required weight reduction for a particular woman (249). Recently, researchers have attempted to circumvent such shortcomings through unimolecular polypharmacy. For example, Finan et al have developed a stable GLP-1-estradiol conjugate (245). They found that a stable GLP-1-estradiol conjugate caused greater weight loss in obese male mice than either GLP-1 controls or labile GLP-1-estradiol conjugates. In labile conjugates, the estradiol rapidly disseminates throughout the circulation in an untargeted fashion. Thus, conjugation appears key to improving GLP-1 and estradiol synergistic effects on weight. The weight loss achieved through the stable conjugate was mainly due to appetite suppression and a decrease in food-intake. Glycemic control and insulin sensitivity were also improved.

Promisingly, this conjugate did not have off-target effects in female mice. Neither the GLP-1 control nor the stable conjugate resulted in an increased uterine weight in ovariectomized mice. This finding indicates that side effects as endometrial hyperplasia would not be expected if administered to women. By contrast, the labile conjugate, which rapidly degrades to release estradiol, did result in increased uterine weight, suggesting off-target effects. Furthermore, LH and FSH levels in mice treated with the GLP-1-estradiol conjugate were unchanged. Thus, the conjugate did not appear to interfere with the hypothalamic-pituitary-gonadal axis.

The authors suggest that the GLP-1-estradiol compound allows targeted delivery of estradiol to the CNS. Thus, conjugate estradiol delivery differs significantly from peripheral administration of estradiol as in HRT for example. Indeed, when mice lacking GLP-1 receptors in the CNS were given the GLP-1-estradiol analogue, weight loss was equivalent only to that of GLP-1 administered peripherally. The conjugate also had beneficial effects on glucose homeostasis. This could be due to an additional effect on hepatic glucose production (245). Furthermore, in New Zealand obese mice, the GLP-1-estradiol conjugate protects against carbohydrate-induced hyperglycemia (250). This was largely due to it causing a reduction in appetite, mediated by an induction of POMC expression. Although peripheral effects of the estradiol-GLP-1 conjugates are anticipated, most evidence suggests that the conjugate acts centrally to suppress food-reward (250). These proof-of-concept studies raise interesting possibilities for therapeutic strategies in humans. There is no research, to the authors’ knowledge, investigating GLP-1 analogs in the postmenopausal population specifically. However, in a recent meta-analysis showing the beneficial effects of GLP-1 analogs on weight loss, over 57% of participants were female, and the median age at randomization was 55 years (233). Given that the average age at menopause is 51 years, it is highly likely that a significant number of participants were peri- or post-menopausal women. Furthermore, given that other anti-diabetic treatments such as SGLT-2 inhibitors are feared to adversely affect bone health, there is increasing interest in the benefits of GLP-1 analogs for diabetic post-menopausal women (251). Given the neuroprotective effects of both GLP-1 and estradiol, it would also be interesting to know how such agents might reduce neurodegeneration, especially in menopausal women.

## Conclusions and future directions

Obesity in women is a global problem. The comorbidities associated with it decrease their quality and life expectancy. Estradiol is crucial not only in reproductive function, but for the regulation of body weight. It has been showed that normal peri-ovulatory estradiol concentrations have anorexigenic effects. Conversely, stages of a woman’s life, such as adolescence, perimenopause and menopause, that are associated with reduced estradiol, are also associated with weight gain. Conditions, such as pregnancy, in which estradiol levels are high, are a high-risk time where susceptible women are more at risk of developing metabolic comorbidities such as obesity and gestational diabetes. Furthermore, ovulatory dysfunction, such as occurs in PCOS, is associated with weight gain and insulin resistance. Estradiol regulates body weight by decreasing appetite and increasing feelings of satiety. Estradiol controls appetite by acting at specific hypothalamic nuclei, such as the ARC or LH. Estradiol also interacts with peripherally synthetized peptides, such as CCK, leptin and insulin. One of these mediators, GLP-1, acts similarly to and, potentially, synergistically with estradiol. Although GLP-1 analogs were initially characterized as antidiabetic agents, they are increasingly being recognized as anti-obesity agents. The reduction in weight gain when GLP-1 analogs are administered is partially explained by their effects on the CNS. The synergistic effects of GLP-1 analogs combined with estradiol conjugates are promising. If translated to human studies, such conjugates could help women to maintain a healthy body weight and preserve their mental function. This could be particularly important for women whose estradiol levels are abnormal, perhaps as a result of ovarian dysfunction, or whose estradiol levels drop, as part of the normal estradiol decline during menopause. GLP-1-estradiol analogues could perhaps be used in the future to improve central insulin sensitivity. As central insulin resistance appears to be a risk factor for neurodegenerative disorders, these analogues might provide interesting avenues to protect against neurodegeneration and conditions such as Alzheimer’s and Parkinson’s disease. Considering the above, future lines of research should focus on the proper dose, timing, safety and frequency when administering GLP-1 analogs and conjugated estradiol as a treatment for body weight disorders.

## Author contributions

PV, JM, GP, and JDR: bibliographic search, writing, review and/or revision of the manuscript. All authors contributed to the article and approved the submitted version.

## Conflict of interest

The authors declare that the research was conducted in the absence of any commercial or financial relationships that could be construed as a potential conflict of interest.

## Publisher’s note

All claims expressed in this article are solely those of the authors and do not necessarily represent those of their affiliated organizations, or those of the publisher, the editors and the reviewers. Any product that may be evaluated in this article, or claim that may be made by its manufacturer, is not guaranteed or endorsed by the publisher.
